# Right Ventricular Perforation by Fractured Sternal Wires: A Narrative Review

**DOI:** 10.21470/1678-9741-2023-0461

**Published:** 2024-10-14

**Authors:** Carlos Gallego-Navarro, Omar Latif, Sorin V. Pislaru, Lawrence J. Sinak, Kevin L. Greason, John M. Stulak, Arman Arghami

**Affiliations:** 1 Department of Cardiovascular Surgery, Mayo Clinic, Rochester, Minnesota, United States of America; 2 Department of Cardiology, Mayo Clinic, Internal Medicine, Rochester, Minnesota, United States of America; 3 Department of Division of Cardiovascular Diseases, Mayo Clinic, Rochester, Minnesota, United States of America

**Keywords:** Bone Wires, Sternum, Heart Injuries, Penetrating Trauma, Postoperative Complications, Incidental Findings.

## Abstract

**Introduction:**

Migration of a fragmented sternal wire is an unusual and rare phenomenon following cardiovascular surgery. It can present with variable clinical presentations, ranging from incidental findings to hemodynamic instability. Here, we described two cases of fragmented sternal wire migration to the right ventricle.

**Methods:**

Retrospective review of the clinical course of two patients presenting with a fragmented sternal wire embedded in the right ventricle after sternotomy for cardiovascular surgery. We also conducted a literature review to identify similar cases, compared them based on reported clinical variables, and discussed the role of diagnostic imaging and management.

**Results:**

We identified 13 patients (11 from the literature), of which 85% were men, and the median age was 64 years; 46% presented with hemorrhagic shock, another 46% had other cardiovascular symptoms, and 8% were asymptomatic. The presentation was bimodal, 54% presented within three weeks of the original sternotomy, while 46% had sternotomy more than a year before. Sternal dehiscence/instability was observed in 61% of cases. Computed tomography scan was the most common diagnostic modality (54%). Two patients did not undergo surgery, and two others died after surgery, while others had a successful surgical repair.

**Conclusion:**

Migration of a fragmented sternal wire is a phenomenon presented on a dehisced and unstable sternum that can occur days or years after sternotomy. These findings and the associated cardiac injury can be easily missed on computed tomography scan reporting if one is not looking for it. After diagnosis, treatment should be individualized according to the patient’s needs.

## INTRODUCTION

**Table:** 

Abbreviations, Acronyms & Symbols				
CABG	= Forced expiratory flow		IQR	= Interquartile range
CCTA	= Cardiac computed tomography angiography		NR	= Non-reported
CPR	= Cardiopulmonary resuscitation		OR	= Operating room
CT	= Computed tomography		POD	= Postoperative day
CTA	= CT angiogram		RV	= Right ventricle
CV	= Cardiovascular		SOB	= Shortness of breath
CXR	= Chest X-ray		TTE	= Transthoracic echocardiogram
HF	= Heart failure		TVR	= Tricuspid valve regurgitation

Fragmentation of a sternal wire is a frequent radiographic finding that usually does not represent a major threat. Its migration is a rare and unusual phenomenon. Even so, migration to many anatomical locations, including the great vessels, vital organs such as the heart and lungs, and other extra-thoracic structures, either by tissue penetration or intravascular embolization, has been described^[1^-^3]^. Each has a varied clinical presentation day to years after sternotomy. Its clinical presentation varies from asymptomatic or benign symptoms, such as chest pain, shortness of breath, palpitations, etc., to hemodynamic instability due to hemorrhagic shock. No comprehensive review or guidelines for diagnosis or management of this condition is currently available. Here, we reported two cases at our institution that presented with a fragmented sternal wire embedded in the right ventricle (RV) after median sternotomy for cardiovascular surgery and a review of 11 more cases from the literature on fragmented sternal wire migration to the RV after sternotomy for adult cardiothoracic surgery.

## METHODS

Written informed consent was obtained from the patient and/or family for publication of the details of their medical case. To gain insight from previous cases, we conducted a literature review using PubMed and Scopus with the keywords (“cardiac” OR “heart”) AND (“sternal wire”). We only included articles in English with sufficient data to enable data abstraction to make comparisons between our cases and those identified in the literature. We identified eleven cases of fragmented sternal wire migration to the RV and compared them by examining common variables that were reported, including sex, age (in years), time of most recent sternotomy, clinical presentation, sternal dehiscence/stability, imaging modality used for diagnosis, management, and outcome.

## RESULTS

### Patient One

An 82-year-old male presented to the emergency department with worsening chest pain around his dehisced sternum. On the initial examination, he was hemodynamically stable with tenderness on the anterior chest.

One year prior, he underwent open mitral valve replacement for severe valvular disease, coronary artery bypass grafting (CABG) from the left internal mammary to the left anterior descending artery, and left atrial appendage ligation. Due to poor bone quality, his sternum was closed with a combination of stainless-steel wires and titanium plates. His history is also significant for atrial fibrillation, chronic kidney disease, obesity, and hypothyroidism.

**Fig. 1:**
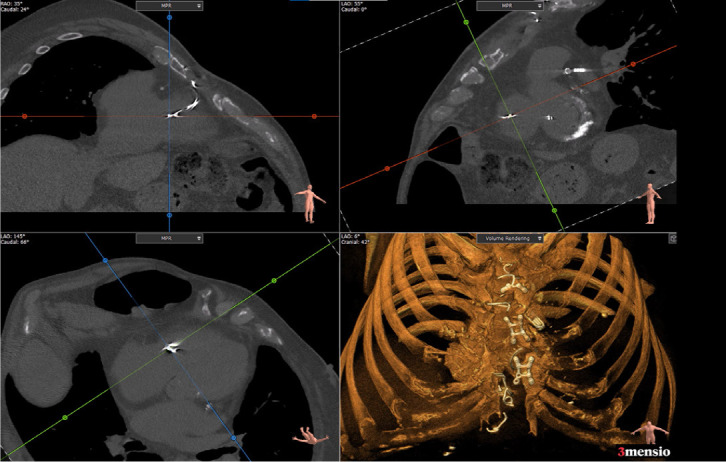
Multiplane non-contrast chest computed tomography: fragmented sternal wire perforating right ventricular wall.

**Fig. 2:**
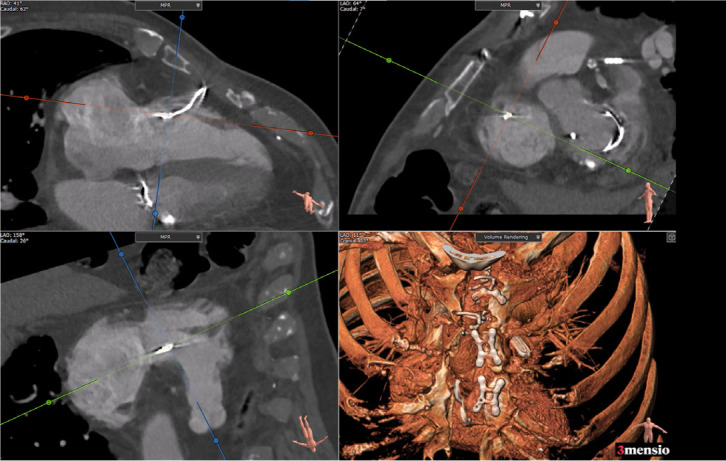
Multiplane chest computed tomography angiogram: fragmented sternal wire perforating right ventricular wall.

**Fig. 3:**
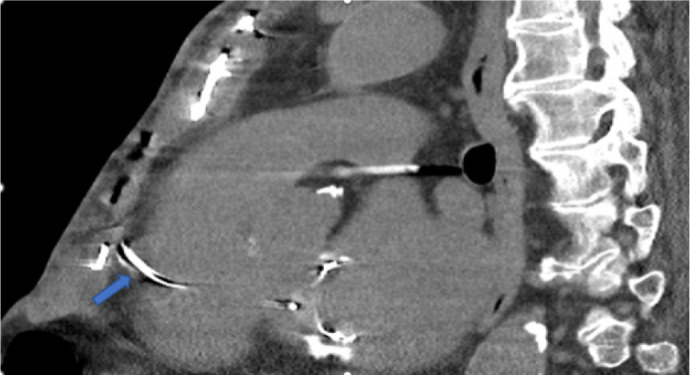
Non-contrast chest computed tomography: sagittal view, demonstrates sternal wire (blue arrow) puncturing basal anterior right ventricular free wall.

**Fig. 4:**
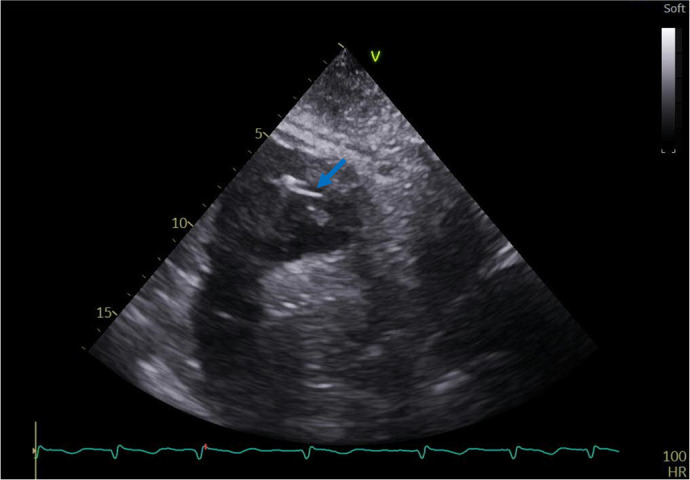
Transthoracic echocardiogram: subcostal view showing the sternal wire (blue arrow) within the right ventricle cavity.

**Fig. 5:**
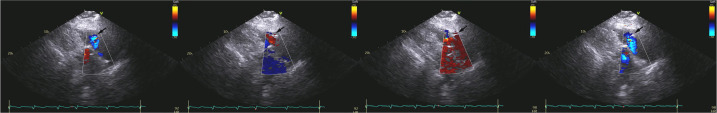
Transthoracic color Doppler echocardiogram: subcostal view tricuspid regurgitation starting at the wire (black arrows).

Seven months and three and a half months before this presentation, a computed tomography (CT) of the sternum and a chest CT angiogram (CTA) at an external site were conducted for sternal dehiscence and pulmonary embolism, respectively, at his local facility; retrospective review of the studies shows a penetrating wire in the heart (Figures 1 and 2), but this was missed on the report. Two weeks prior right ventricular perforation was suspected on echocardiography, and a chest CT showed a stable sternal dehiscence and a fractured sternal wire partially perforating the free wall of the RV (Figure 3), so removal of the sternal wire was scheduled. On admission, a cardiac CTA proved that the tip of the fractured wire in the RV was puncturing its basal anterior free wall and abutting the tricuspid valve. This was limiting the mobility of the tricuspid leaflets resulting in severe tricuspid valve regurgitation (TVR) demonstrated by transthoracic echocardiogram (TTE) (Figures 4 and 5).

Although he was hemodynamically stable, the concerning imaging and worsening chest tenderness prompted the urgent removal of the fragmented wire under fluoroscopy guidance. The previous sternotomy incision was reopened, and a pocket of sterile fluid was encountered with fragmented wires and loose plates floating within it. The free end of the broken standard stainless-steel wire was mobile with each heartbeat (Supplementary Video). This was removed carefully from the right ventricular free wall without the need for cardiopulmonary bypass under fluoroscopy guidance and control. The entry site was closed with a 4-0 non-absorbable monofilament pledged suture. The edges of the sternum were completely fused to the underlying mediastinum with a thick scar. Therefore, the rest of the wires, plates, and screws, which were all loose and broken, were removed. Intraoperative transesophageal echocardiogram post sternal wire removal reported residual severe TVR with central and eccentric anteriorly directed jets. After evaluating the case, due to its non-urgent nature and no hemodynamic effects from the TVR, valve replacement and sternal re-fixation were not pursued. The patient had an uneventful postoperative course and was discharged eight days after surgery. He completed rehabilitation in a skilled nursing facility and is scheduled for a follow-up echocardiogram.

**Supplementary Video:**
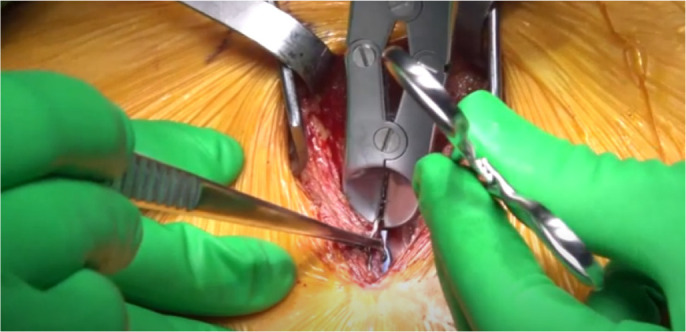
Extraction of a fragmented sternal wire embedded in the right ventricle, mobile with each heartbeat.

Link: https://youtu.be/1cA0hTK8TqA

### Patient Two

A 66-year-old male presented to the emergency department with a three-day history of progressive shortness of breath and a warm erythematous lesion over his lower chest and a bloody drainage from a lower sternal incision.

Three weeks before, he underwent bioprosthetic aortic valve replacement for calcific aortic stenosis which was complicated with postoperative sternal wound bleeding and anemia requiring transfusion of blood products, one week after surgery. His medical history included coronary artery disease, status post-bare metal stent to the left anterior descending artery a year before, non-alcoholic steatohepatitis, cirrhotic liver disease with early-stage hepatocellular carcinoma, severe coagulopathy, and thrombocytopenia. It was also significant for mitral valve endocarditis due to *Staphylococcus aureus* in 2005, insulin-requiring type 2 diabetes mellitus, obesity, and obstructive sleep apnea.

On admission, an electrocardiogram reported low voltage and a TTE demonstrated a large pericardial effusion, confirming the diagnosis of pericardial tamponade in a patient with tamponade physiology. The patient underwent pericardiocentesis with drainage of 1.7 L of bloody fluid with rapid reaccumulation of pericardial fluid intraoperatively. Fluid removal was held, and the patient was admitted to the cardiac care unit for monitoring. A few hours later, TTE demonstrated reaccumulation of fluid and a second pericardiocentesis was performed from a different window and drained 750 mL of bloody fluid. Blood, wound, and pericardial fluid cultures all returned positive for coagulase-negative *Staphylococcus*, and antibiotic therapy was initiated.

**Fig. 6:**
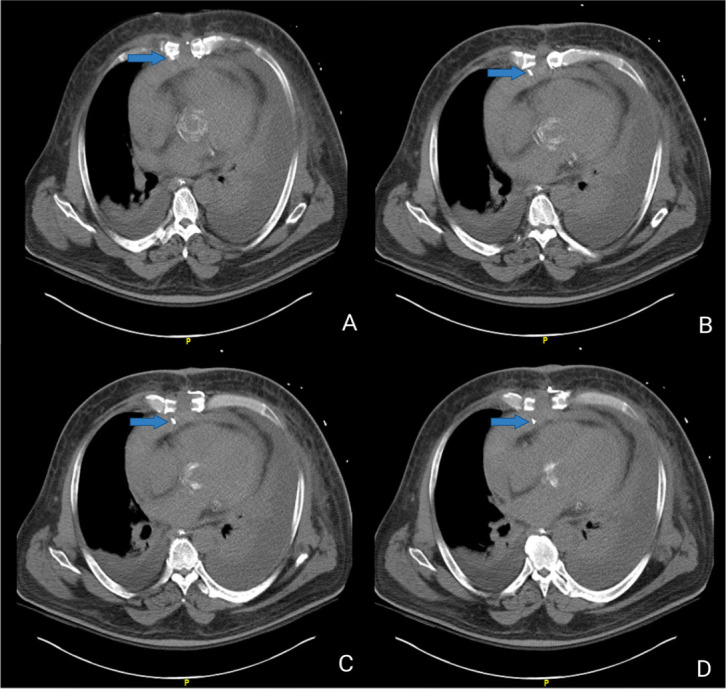
Non-contrast chest computed tomography: axial view, sequence of figures (A, B, C, D) showing broken sternal wire (blue arrows) puncturing pericardial sac over the right ventricular free wall.

Given the suspicion of sternal infection, a chest CT scan was conducted; it revealed dehiscence of the lower sternotomy with multiple broken sternal wires, but no pericardial wire perforation was reported until reviewed and recognized by our clinical team (Figure 6) due to non-hemodynamic improvement and a subsequent TTE reporting significant pericardial effusion around the right heart chambers with right atrial compression. We hypothesized that the initial effusion was due to wire perforation of the RV and/or infection/inflammatory reaction. As the effusion enlarged, the increased space between the sternum and RV allowed the wire to exit the right ventricular cavity and stop the initial intrapericardial bleeding. At the first pericardiocentesis, removing the fluid resulted in repeat impalement of the RV on the broken wire with repeat intrapericardial bleeding being responsible for rapid fluid reaccumulation. A Swan-Wanz hemodynamic catheter was placed to assist in diagnosis and revealed high right heart pressures. After deliberating, the patient was taken to the operating room, where he was found to have sternal dehiscence with mediastinal hemorrhage and infection of the dehisced sternotomy. He had multiple sternal bones and wire fractures with a right ventricular tear. He underwent sternectomy, removal of the sternal wires, debridement, and irrigation multiple times in the following days, a muscle flap was used for closure.

His postoperative course was critical, and he remained admitted to the surgical intensive care unit with increasing systemic inflammatory response. He developed new mitral valve vegetation not evident on prior echocardiograms. The patient required increased hemodynamic and antibiotic support but ended up developing multiorgan failure. Given his clinical status decline, non-healing chest wound, and new mitral valve vegetation, the patient expired on postoperative day 17 after the family agreed to provide comfort care alone.

All findings from each case we found in the literature and a descriptive analysis of the data are summarized in Tables 1 and 2.

**Table 1:** Characteristics of patients with fragmented sternal wire migration to the right ventricle.

Variable, n = 13	n (%)
Sex, male	11 (85)
Age in years, median (IQR)	64 (60, 64)
Time of recent sternotomy	
Less than a week	3 (23)
Two to three weeks	4 (31)
More than one year	6 (46)
Clinical presentation	
Any CV symptom (chest pain, SOB, palpitations, cough, etc.)	6 (46)
Any form of CV symptom + hemorrhagic shock	6 (46)
Asymptomatic	1 (8)
Sternal instability/dehiscence	
Dehiscence	8 (61)
Stable sternum	1 (8)
No reported	4 (31)
Imaging modality	
CT, CCTA +/- CXR, echocardiogram	7 (54)
CXR +/- echocardiogram	3 (23)
None	3 (23)
Management	
Surgical	11 (85)
Conservative or expectant	2 (15)
The outcome, alive at discharge, and follow-up	11 (85)

CCTA=cardiac computed tomography angiography; CT=computed tomography; CV=cardiovascular; CXR=chest X-ray; IQR=interquartile range; SOB=shortness of breath

**Table 2:** Reported cases of fragmented sternal wire migration to the right ventricle.

Author, year	Age, sex	Most recent sternotomy	Presentation	Sternal instability/dehiscence	Imaging modality before definitive management	Surgery	Outcome
Gallego (patient 1)	82/male	1 year	Worsening chest pain	Yes	Sternal CT and chest CTA (for different reasons months prior), chest CT	Yes	Discharged on POD 8
Gallego, (patient 2)	66/male	3 weeks	Progressive SOB, bloody drainage from the sternal incision, and shock	Yes	Chest CT	Yes	Death on POD 17
Chang, 1989^[13]^	57/male	24 days	Severe cough, chest pain, massive bleeding from incisions, and shock	Yes	None, transported immediately to the OR	Yes	Intraoperative death after CPR
Al Halees, 2007^[14]^	67/male	10 years	HF symptoms	NR	Lateral CXR	Yes	NR
Levisman, 2010^[6]^	64/male	7 years	Asymptomatic (preoperative screening of ventral hernia repair)	Yes	CXR, coronary angiogram, CCTA	Yes	Stable and healed sternum at 6-month follow-up
Rungatscher, 2012^[5]^	68/male	7 days	Hypotension, tachycardia, and increased central venous pressure, and shock	NR	None	Yes	Complete recovery
Shukla, 2012^[15]^	61/male	17 days	Severe cough, chest pain, massive bleeding from incisions, and shock	Yes	Chest CT	Yes	Discharged on POD 9
Gong, 2015^[2]^	60/male	3 days	Severe cough, massive chest bleeding, and shock	NR	None, transported immediately to the OR	Yes	Discharged on POD 8
Abdul Ghani, 2016^[3]^	56/male	6 years	Sharp, dull pain left-sided chest pain, and dyspnea	No	CXR, coronary angiogram, chest CT^*^	No^¶^	Left against medical advice
Efthymiou, 2019^[4]^	52/male	3 weeks	Minor bleeding from incisions	Yes	CXR	Yes	Discharged after rehabilitation
Abe, 2020^[9]^	63/female	17 years	Palpitations	NR	CXR^*^, ECG-synchronized CT, echocardiogram	No (conservative management)	Stable wire at 2-year follow-up
Ethier, 2020^[7]^	68/female	Several years	Recurrent SOB	Yes	CXR, coronary angiogram, chest CT	Yes	Discharged on POD 1
O’Brien, 2022^[12]^	68/male	6 days	Sudden SOB, bleeding from sternal incisions, and shock	Yes	CXR, bedside echocardiogram	Yes	Discharged on POD 15

CCTA=cardiac computed tomography angiography; CPR=cardiopulmonary resuscitation; CT=computed tomography; CTA=CT angiogram; CXR=chest X-ray; HF=heart failure; NR=non-reported; OR=operating room; POD=postoperative day; SOB=shortness of breath

* Conducted on several occasions over the years;

¶ Patient refused treatment and left against medical advice

## DISCUSSION

In this study, we described the clinical course of two patients who were presented at our institution and compared them to eleven previous similar reports in the literature. Our analysis of the 13 patients revealed that the most common presentation was cardiovascular symptoms including chest pain, palpitations, and shortness of breath among others but without significant bleeding in 46% of the patients, whereas in 46% of patients, in addition to cardiovascular symptoms, incisional bleeding or hemorrhagic shock occurred, and the remaining 8% were asymptomatic. Notably, all six patients who had hemorrhagic shock presented less than three weeks after the initial surgery, and only the case described by Efthymiou et al.^[4]^, who presented before three weeks, presented with minor bleeding without shock.

Five out of seven patients who presented less than three weeks after the initial sternotomy were found to have sternal dehiscence/instability. For the patients described by Rungatscher et al.^[5]^ and Gong et al.^[2]^, although not explicitly reported, it is plausible to assume that complete sternal stability was not achieved either, likely due to the short healing time since initial surgery. It is also noteworthy that the remaining three cases with sternal dehiscence presented one year after their initial surgery where complete healing of the sternum is expected (Levisman et al.^[6]^, Ethier et al.^[7]^) (*patient no. 1*). In a literature review, Mokhtar et al.^[1]^ described eleven patients with sternal wire migration to structures mostly other than the RV, all but one patient presented more than one year after the initial sternotomy and half of them had a dehiscent sternum.

The sternum takes an average of three-six weeks to heal 80%, and complete healing is not reached until three months after a median sternotomy for CABG^[8]^. Most wire migration occurs years after sternotomy in a dehisced and improperly healed sternum, likely due to repetitive motion related to sternal nonunion. Routine follow-up including chest radiography is crucial and should be considered for early detection of sternal instability, wire cracking, and fragmentation in suspected patients^[9]^.

To identify the migrated fragmented sternal wire to the heart, our review showed that the CT scan either alone or with additional imaging was the most reported tool in 54% of the patients, the remaining 46% did not require it (Table 1), as the patients were rushed immediately to the operating room or the wire was promptly identified with other modalities. Chest CT scans have reported a sensitivity of over 76.9% and a specificity of 99.7% for detecting hemopericardium in penetrating cardiac lesions^[10]^. The chest CT scans of our two patients revealed heart perforation caused by a fragmented sternal wire. However, in *patient no. 1*, both initial CT scans were conducted for varied reasons and the perforation went unnoticed. In both instances, the lack of an open perspective and systematic reading of the CT and the unawareness of the likelihood of incidental findings most likely led to the lesion not being reported and so a delay in the definitive diagnosis and management. With the continued rise in advanced imaging, radiologists interpreting chest CT will diagnose incidental cardiac disease, injuries, or artifacts, therefore radiologists and the clinical team should be aware of any appearances different from what is being expected, as some may require further studies and or management so that they can direct further appropriate management^[11]^.

To manage these patients, emergent surgery should be performed in critical cases, such as pericardial tamponade^[12]^. *Patient no. 2* presented with pericardial tamponade and, despite undergoing an emergency CT scan, the perforation of the wire to the heart was not detected. As a result, multiple taps were performed on the patient, which unfortunately led to further injuries to the RV before the definite wire removal surgery was conducted. In *patient no. 1*, although initial CT scans were performed for varied reasons and did not identify the perforation, later comprehensive analysis that included 3-D reconstruction helped determine the relative position of the fractured wires in the chest cavity, particularly in contrast studies.

Incidental findings of fractured sternal wire should be reviewed carefully by cross-sectional imaging. Factors such as time from the initial surgery, stability of the sternum, and location of the wire can help the decision-making for the providers to intervene on fractured sternal wires before they create a potential cardiac trauma such as bleeding, tamponade, or valvular injury (such as in *patient no.1*). The fractured wire can dislodge and move with every heartbeat that can increase morbidity and mortality and require urgent attention^[6]^.

It is worth mentioning that although one of these cases occurred in one patient (*patient no. 1*) whose sternum was closed with a combination of stainless-steel wires and titanium plates, we do not have any evidence indicating a correlation between sternal plating system and the event. In fact, in this case the perforation occurred with a standard stainless steel sternal wire. The fact that the sternal plating was used in this patient only shows that he was assumed to be at risk of sternal non-union and complication, which had then resulted in wire breaking and puncturing the RV.

### Limitations

The limitation of this study is the retrospective data that may limit the generalizability and the robustness of the findings. Furthermore, inconsistencies in how similar cases are reported in the literature could affect the comparisons' reliability.

## CONCLUSION

In conclusion, migration of a fragmented sternal wire is a rare phenomenon presented on a dehisced and unstable sternum that can present days or years after sternotomy. These findings and the associated cardiac injury can be easily missed on CT scan reports if one is not looking for it. After diagnosis, treatment should be individualized according to the patient's needs.
